# Impaired left and right systolic ventricular capacity in corrected atrial septal defect patients

**DOI:** 10.1007/s10554-021-02506-7

**Published:** 2022-02-07

**Authors:** Zarmiga Karunanithi, Mads Jønsson Andersen, Søren Mellemkjær, Mathias Alstrup, Farhad Waziri, Tor Skibsted Clemmensen, Vibeke Elisabeth Hjortdal, Steen Hvitfeldt Poulsen

**Affiliations:** 1 Department of Cardiothoracic and Vascular Surgery, Aarhus University Hospital, Palle Juul-Jensens Boulevard 99, 8200 Aarhus N, Denmark; 2 Department of Cardiology, Aarhus University Hospital, Palle Juul-Jensens Boulevard 99, 8200 Aarhus N, Denmark; 3 Department of Clinical Medicine, Aarhus University, Palle-Juul Jensens Boulevard 82, 8200 Aarhus N, Denmark; 4 Department of Cardiothoracic Surgery, Rigshospitalet, University of Copenhagen, Blegdamsvej 9, 2100 Copenhagen Ø, Denmark

**Keywords:** Atrial septal defect, Congenital heart defect, Stress echocardiography, Cardiopulmonary exercise test

## Abstract

Resting right ventricular (RV) systolic function has in some studies been shown to be impaired after correction of an atrial septal defect (ASD) whereas impairment of left ventricular (LV) systolic function is uncertain. In the present study we examine the LV and RV systolic response to exercise in patients with a previously corrected ASD in order to investigate the myocardial capacity. Thirty-six adult ASD patients with a corrected isolated secundum ASD and eighteen adult age-matched controls underent a semi-supine exercise stress echocardiographic examination. At rest, LV parameters were comparable between groups, and RV global longitudinal strain (RV-GLS) was lower for the ASD group (−18.5%, 9﻿5% CI −20.0—−17.0%) compared with controls (−24.5%, 95% CI −27.7—−22.4%, *p* < 0.001). At peak exercise, LV ejection fraction (LVEF) was lower for ASD patients (61%, 95% CI 58–65%) compared with controls (68%, 95% CI 64–73% *p* = 0.01). Peak LV global longitudinal strain (LV-GLS) was borderline significantly lower (ASD: -18.4%, 95% CI −20.2—−16.6%, controls: −21.3%, 95% CI −23.6—−19.0%, *p* = 0.059). Both RVEF (ASD: 64%, 95% CI 60–68%, controls: 73%, 95% CI 65–80%, *p* = 0.05) and tricuspid annular plane systolic excursion (TAPSE) (ASD: 2.5 cm, 95% CI 2.3–2.7 cm, controls: 3.2 cm, 95% CI 2.9–3.6 cm, *p* < 0.001) at peak exercise were lower for ASD patients. Exercise assessed peak oxygen uptake was comparable between groups (ASD: 32.8 mL O_2_/kg/min, 95% CI 30.3–35.5 mL O_2_/kg/min, controls: 35.2 mL O_2_/kg/min, 95% CI 31.6–38.8 mL O_2_/kg/min, *p* = 0.3). Corrected ASD patients demonstrate a reduced LV and RV systolic exercise response decades after ASD correction whereas resting parameters of LV and RV systolic function were within normal range. The presence of subclinical systolic myocardial dysfunction during exercise might be associated with the long-term morbidities documented in this patient group.

**Supplementary Information:**

The online version contains supplementary material available at 10.1007/s10554-021-02506-7.

## Introduction

Uncorrected atrial septal defects (ASD) cause volume overload of the right ventricle and pulmonary circulation with the risk of developing myocardial dysfunction and pulmonary vascular remodeling. Once corrected, the right-sided volume overload is mitigated and right atrial (RA) and ventricular (RV) remodeling occur leading to reduced chamber dimensions, while the left-sided volume flow and left ventricular (LV) dimensions increase [1–3]. LV systolic function seems unaffected by the ASD correction as LV ejection fraction (LVEF) and global longitudinal strain (GLS) are unchanged and within normal range after ASD correction in adult patients [4–6]. Interestingly, RV systolic impairments appear present after ASD correction as fractional area change (RV FAC), tricuspid annular plane systolic excursion (TAPSE), and RV systolic velocity (RV-s′) are reduced [4, 7]. Deformation analysis may also indicate subtle RV systolic dysfunction than conventionally reported volumes and ejection fraction. RV global longitudinal strain (RV-GLS) gradually improves during the first 3 months after ASD correction [5, 8]. Impaired RV-GLS values have been reported in children months after percutaneous ASD closure [6]. Even though there is no current official guideline consensus of normal reference values for RV-GLS, normal range values has been suggested by Muraru et al.[9] with a lower limit of −20% for adults.

Removing the volume overload seems to improve specific systolic myocardial function parameters in the months following ASD correction. Yet, for unexplained reasons, long-term morbidity and early mortality are still increased [10–12]. Evaluating RV and LV systolic function at rest may not adequately describe the myocardial performance. Similar to how Bonow et al.[13] revealed an abnormal LVEF response to exercise in uncorrected ASD patients, stress testing could provide additional knowledge and reveal subclinical myocardial dysfunction in corrected ASD patients. We hypothesize that adult ASD patients have decreased biventricular systolic function at peak exercise compared with healthy controls. This study aims to determine if adult ASD patients have signs of impaired myocardial performance long-term (decades) after ASD correction evaluated by a comprehensive physiological stress echocardiographic examination. Multiple studies have demonstrated the benefits of ASD correction as cardiac function improves, primarily at rest [1–6, 14]. The purpose of this study is not to prove what is already known, but rather to add another aspect, as we focus on the ventricular functional performance during physical stress after atrial septal defect correction.

## Materials and method

### Study population

**Fig. 1:**
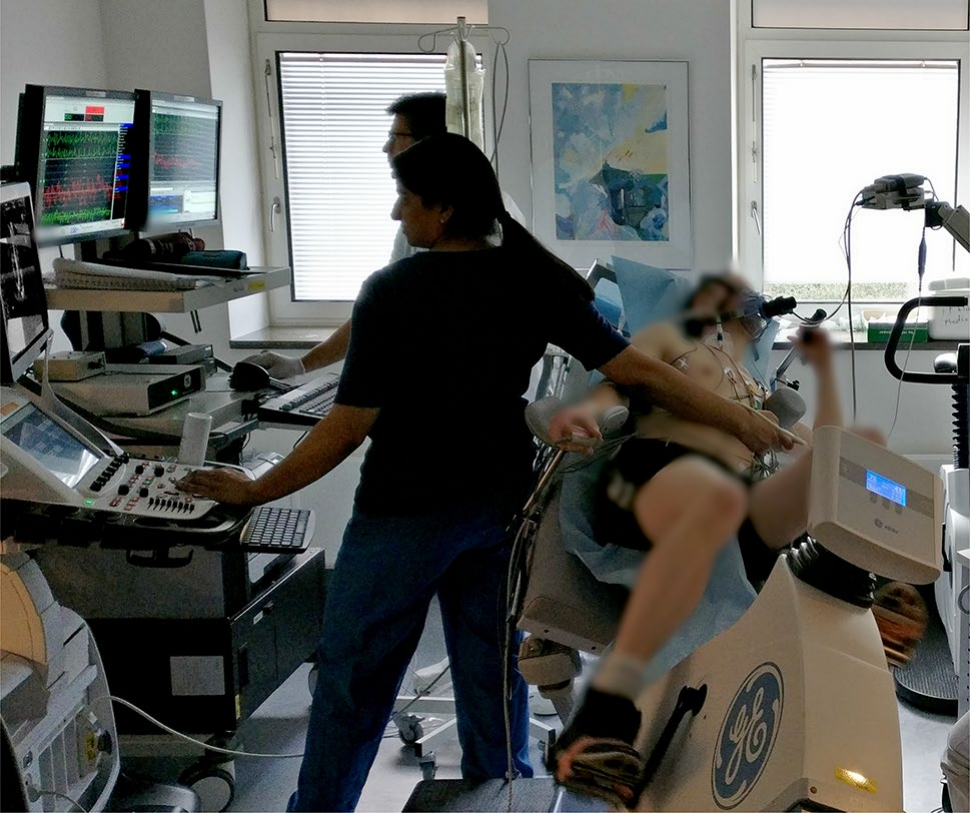
Examination setup. An image of the examination setup. The participant is installed on a semi-supine ergometer wearing a mask to measure direct oxygen consumption. Heart rate, saturation and blood pressure are constantly monitored. The cardiopulmonary exercise test is simultaneously performed with a stress echocardiographic examination and invasive hemodynamic measurements through an already placed Swan Ganz catheter. The participant exercises until maximal exhaustion with work load increments every three minutes. Stress echocardiographic images and pressure measurements were obtained during the last 1.5 min of each 3-min load increment

**Fig. 2:**
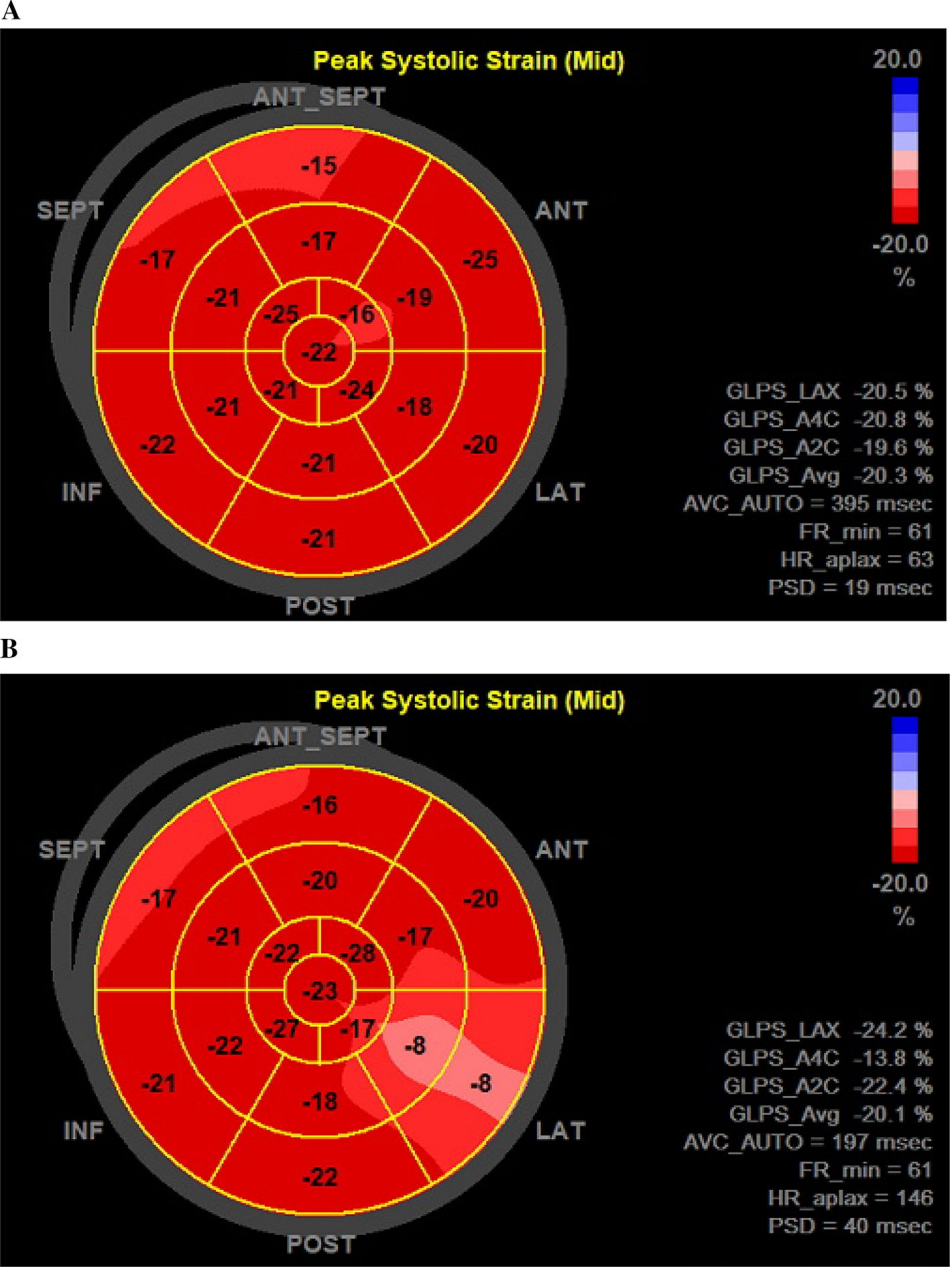
An example of a bulls eye plot of the left ventricular global longitudinal strain for the same ASD patient at rest (**A**) and peak (**B**) exercise

The study included 38 adults with a corrected isolated secundum ASD (19 surgical, 19 percutaneous) and 19 age-matched healthy controls. All underwent a semi-supine exercise stress echocardiographic examination (Figs. 1 and 2, videos 1–3) from August 2018 to October 2019 at Aarhus University Hospital, Denmark. Clinical databases at Aarhus University Hospital, Denmark, were used to identify ASD patients. To be eligible, the patients had to be at least 2 years old when diagnosed with the ASD, and more than 3 years had to have passed since their ASD correction. Controls from the background population were found through public announcement and were eligible if they had no cardiopulmonary diagnoses, did not use prescription medicine interfering with their cardiopulmonary function, and had a normal electrocardiogram and echocardiography. All participants were minimum 18 years when examined.

Two ASD and one control participant were excluded as one ASD patient had breast implants that did not allow for a suitable acoustic window during stress echocardiography, one ASD patient did not undergo stress echocardiography due to a vasovagal episode, and one control did not perform stress echocardiography as examinations were terminated prematurely due to a brief self-limiting period of atrial fibrillation.

None of the ASD patients nor controls had pulmonary hypertension, as mean pulmonary artery pressure at rest was 14 ± 3 mmHg for ASD patients and 15 ± 2 mmHg for controls, or exercise-induced pulmonary hypertension [15].

### Echocardiography

Baseline transthoracic echocardiography was performed according to current guidelines on a Vivid E95 ultrasound unit (GE Healthcare, Horten, Norway) using the M5S and 4 V-D probes to obtain 2D and 3D images for analysis [16]. Stress echocardiography was performed using the M5S (2D imaging) transducer with the participant on a semi-supine ergometer with a left lateral tilt for an optimal acoustic window simultaneously with a cardiopulmonary exercise test (CPX) with expired gas analysis.

Apical 4-, 3-, and 2-chamber views of the LV and a modified 4-chamber view of the right ventricle (RV) and right atrium (RA), both with and without tissue Doppler imaging, were obtained. LV volumes and ejection fraction (LVEF) were calculated using the biplane disc method. RV volumes and ejection fraction (RVEF) were also calculated using method of disks. Left atrial (LA) and RA volume traced in the 4-chamber apical view at the largest volume were indexed to body surface area (BSA). RV fractional area change (FAC) was calculated as (RV end-diastolic area—RV end-systolic area)/RV end-diastolic area.

Mitral valve E velocity was measured by pulsed wave Doppler at the tip of the mitral valve. LV s′ (LV-s′), e′ (LV-e′) and a′ (LV-a′) wave velocities were measured in the lateral mitral annular segment using color tissue Doppler imaging. Peak systolic tricuspid annular velocity (RV-s′) was measured in the lateral tricuspid annulus using tissue Doppler imaging. TAPSE was measured in the lateral tricuspid annulus on a 4-chamber apical view with M-mode.

LV-GLS was calculated by speckle tracking the LV in all three apical views (18 segments). Images had a frame rate > 55 frames/s. They were analyzed using EchoPAC™’s Automated Function Imaging (AFI) with manual adjustment of the speckles for optimization and the event timing function to define aortic valve closure. RV-GLS was assessed from the modified RV 4-chamber view requiring at least four of six segments for strain analysis, while RV free wall global longitudinal strain (RV-FWGLS) was assessed using the lateral three segments. Images had a frame rate > 55 frames/second and were analyzed using EchoPAC™’s Q-analysis and 2DStrain function.

Images were blinded and analzed offline using EchoPAC™ PC SW-Only version 202 (GE Healthcare, Horten, Norway) by one investigator (ZK), who was blinded to clinical information.

### Cardiopulmonary exercise test

The cardiopulmonary exercise test was performed on a semi-supine ergometer (GE eBike L Ergometer, Freiburg, Germany) with simultaneous expired gas analysis (Jaeger MasterScreen CPX, CareFusion, 234 GmbH, Hoechberg, Germany) enabling directing measurement of oxygen uptake through a mask. The exercise protocol began at 0 W with load increments of either 25 W or 50 W every three minutes based upon the participant’s fitness level. Electrocardiogram, blood pressure, and saturation were continuously monitored. Participants maintained a constant pedaling speed of 60–70 rotations per minute and continued until maximal exhaustion, defined as a respiratory exchange ratio (RER) ≥ 1.1 and Borg > 18 [17]. The study population was exposed to the same level of strenuous activity. Echocardiographic images were obtained during the last 1.5 min of each 3-min load increment.

### Statistical analysis

Sample size for the original study was calculated based upon maximal oxygen uptake (VO_2_) values (the primary endpoint of the main study) from an exercise study on ventricular septal defect patients and controls [18]. With an alpha of 5%, beta of 15%, and a power of 85%, 18 participants needed to be included in each subgroup (controls, surgically corrected ASDs, and percutaneously corrected ASDs).

Results are reported as mean (95% confidence interval) for normally distributed variables, as median (interquartile range, IQR) for non-parametric continuous variables, or as number (%) for categorical variables. Certain non-parametric continuous variables (body surface area, age at correction, left ventricular end-systolic volume, left atrial end-systolic volume index) were log-transformed before statistical analyses if that resulted in a normal distribution. The calculated log(mean) and log(SD) were then transformed back using the exponential function.

Twoway mixed effects analysis of variance (ANOVA) and unpaired Student’s t-tests were used on normally distributed continuous data. The Wilcoxon rank-sum test was used on non-parametric continuous data. Fisher’s exact test was used on binomial data. The significance level was set at 5%.

To asses reproducibility, two investigators (ZK and FW) measured LVEF and TAPSE at rest and peak exercise in 15 randomly selected participants. Both investigators were blinded to the primary results and clinical information and assessed the images > 3 months after the primary assessment. Intra- and interobserver variability were evaluated by calculating the absolute mean differences and estimating the intraclass correlation coefficient (ICC) using the mixed-effects model to report consistency of agreement. Reproducibility of LV-GLS and RVEF have previously been evaluated by our laboratory in 20 and 15 patients, respectively [19, 20].

REDCap electronic data capture tools hosted at Aarhus University, Denmark, was used for data collection and management, and Stata/SE 16.1 (StataCorp LLC, TX, USA) for statistical analyses.[21, 22]

## Results

### Baseline demographics and cardiopulmonary exercise

Thirty-six ASD patients (18 surgically corrected and 18 percutaneously corrected) and 18 controls with a median age of 26 years (range 24–36 years) and 34 years (range 24–43 years), respectively, were evaluated by resting and exercise stress echocardiography.

ASD patients and controls were demographically comparable on all parameters aside from sex (Table 1). Cardiopulmonary exercise parameters (Table 2) were comparable between groups. The mean time from ASD correction to the exercise stress echocardiography was 18 ± 7 years.

**Table 1:** Resting clinical and echocardiographic data

	ASD *n* = 36	Controls *n* = 18	*p* value
Age at examination, years	26 (24–36)	34 (24–43)	0.3
Females, *n* (%)	25 (69)	7 (39)	0.04
BSA, m^2^	1.8 (1.8–1.9)	1.9 (1.8–2.0)	0.4
Age at diagnosis, years	5.5 (1.2–13.1)	–	NA
Age at correction, years	9.7 (7.9–12.1)	–	NA
Valve disease, *n*	0	0	NA
*Echocardiography*			
LV EDV, mL	104 (96–112)	112 (101–123)	0.2
LV ESV, mL	40 (36–45)	43 (38–48)	0.5
LV mass index, g/m^2^ (Devereux formula)	82 (75–89)	77 (68–86)	0.4
LAVi, ml/m^2^	18.4 (16.3–20.9)	19.7 (16.6–23.3)	0.5
E/A	2.2 (2.0–2.4)	1.9 (1.6–2.2)	0.2
E-DT, ms	223 (203–243)	192 (167–217)	0.06
LV-e′, cm/s	12.0 (10.9–13.8)	11.9 (10.7–13.4)	0.7
LV-a′, cm/s	4.3 (3.6–4.9)	4.8 (3.7–5.9)	0.4
RV EDV (3D), mL	95 (87–102)	111 (95–127)	0.03
RV ESV (3D), mL	55 (50–60)	54 (50–60)	1.0
RAVi, mL/m^2^	21.0 (18.8–23.2)	17.8 (15.4–20.3)	0.07

Data are presented as mean (95% cofindence interval), median (interquartile range) or number (frequency). Comparative analyses are performed using Student’s t-test, Wilcoxon rank-sum test, or Fisher’s exact test. *NA* Not applicable, *BSA* Body surface area, *LV EDV* Left ventricular end-diastolic volume, *LV ESV* Left ventricular end-systolic volume, *LV* Mass index, left ventricular mass index, *LAVi* Left atrial end-systolic volume index, *E-DT* Mitral E-wave deceleration time, *LV-e’* Lateral mitral annular e’ wave velocity, *LV-a’* Lateral mitral annular a’ wave velocity, *RV EDV* Right ventricular end-diastolic volume, *RV ESV* Right ventricular end-systolic volume, *RAVi* Right atrial end-systolic volume index. The reported RV volumes are obtained from 3D images

**Table 2:** Cardiopulmonary function

ASD, *n* = 36		Rest	50 W	Peak
Controls, *n* = 18				
Workload	ASD	0	50	167 (151–182)
	Control	0	50	188 (163–212)
	*p* value	NA	NA	0.1
HR, min^−1^	ASD	77 (72–83)	109 (103–115)	175 (169–180)
	Control	70 (63–78)	101 (93–110)	170 163–178)
	*p* value	0.2	0.1	0.3
sBP, mmHg	ASD	141 (134–147)	157 (148–165)	208 (192–223)
	Control	121 (111–132)	151 (137–165)	212 (189–235)
	*p* value	0.003	0.5	0.8
dBP, mmHg	ASD	69 (65–72)	78 (71–86)	93 (80–106)
	Control	70 (64–76)	76 (65–88)	86 (66–105)
	*p* value	0.8	0.8	0.5
VO_2_, mL/kg/min	ASD	4.9 (4.6–5.3)	13.7 (12.6–14.5)	32.8 (30.3–35.3)
	Control	4.6 (4.1–5.0)	13.6 (12.6–14.5)	35.2 (31.6–38.8)
	*p* value	0.2	0.8	0.3
RER	ASD	0.8 (0.8–0.8)	0.8 (0.8–0.9)	1.2 (1.1–1.2)
	Control	0.8 (0.8–0.8)	0.9 (0.8–0.9)	1.2 (1.1–1.2)
	*p* value	0.1	0.4	0.7
Lactate, mmol/L	ASD	0.7 (0.6–0.9)	NA	11.0 (9.9–12.1)
	Control	0.5 (0.3–0.7)	NA	10.5 (8.9–12.1)
	*p* value	0.1	NA	0.6

Data are presented as mean (95% confidence intervals). Comparative analyses are performed using Student’s t-test (for peak workload) or two-way mixed effects analysis of variance. *NA* Not applicable, *HR* Heart rate, *sBP* Systolic blood pressure, *dBP* Diastolic blood pressure, *VO2* Oxygen uptake, *RER* Respiratory exchange ratio

### Left ventricular function

Baseline resting LV (and RV) echocardiographic parameters are presented in Tables 1 and 3.

**Table 3:** Echocardiographic data during exercise

ASD, *n* = 36		Rest	50 W	Peak
Control, *n* = 18				
LV-s′, cm/s	ASD	6.4 (5.7–7.0)	9.3 (8.4–10.3)	12.0 (10.9–13.0)
	Controls	7.7 (6.8–8.7)	10.6 (9.2–11.9)	14.4 (13.0–15.9)
	*p* value	0.02	0.1	0.01
E/e′	ASD	7.3 (6.6–8.1)	8.9 (7.5–10.2)	10.6 (8.0–13.1)
	Controls	6.7 (5.6–7.7)	7.3 (6.1–8.5)	9.0 (7.0–11.1)
	*p* value	0.3	0.1	0.1
RV-s′, cm/s	ASD	8.7 (8.1–9.2)	11.9 (11.1–12.6)	13.4 (12.5–14.3)
	Controls	10.2 (9.0–11.3)	12.2 (10.7–13.7)	15.2 (13.6–16.8)
	*p* value	0.02	0.7	0.07
RV FAC, %	ASD	41.5 (39.1–44.0)	45.4 (42.1–48.7)	49.1 (46.0–52.3)
	Controls	42.9 (38.2–47.5)	52.7 (46.6–58.7)	57.7 (51.9–63.4)
	*p* value	0.6	0.05	0.02
RV-FWGLS, %	ASD	−21.2 (−23.4—−19.1)	−23.2 (−25.3—−21.0)	−20.9 (−23.4—−18.4)
	Controls	−28.0 (−31.1—−24.9)	−20.9 (−25.1— −16.8)	−25.7 (−31.1—−20.2)
	*p* value	0.001	0.4	0.1

Data are presented as mean (95% confidence intervals). Comparative analyses are performed using two-way mixed effects effects analysis of variance. *LV-s*′ Lateral mitral annular s′ wave velocity, *E/e’* Left ventricular diastolic function, *RV- s*′ Lateral tricuspid annular s′ wave velocity, *RV FAC* Right ventricular fractional area change, *RV-FWGLS* Right ventricular free wall global longitudinal strain

**Fig. 3:**
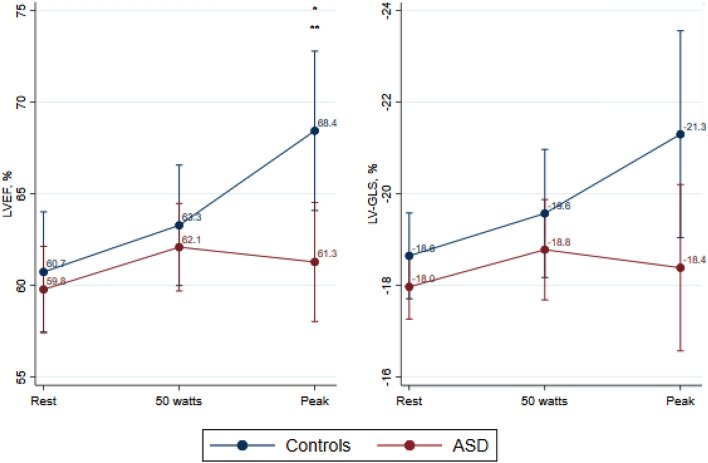
Left ventricular parameters. Mean and 95% confidence intervals at rest, exercise at 50 watts and peak exercise. Comparative analyses are performed using two-way mixed effects analysis of variance. *LVEF* Left ventricular ejection fraction, *LV-GLS* Left ventricular global longitudinal strain. * marks a significant difference (*p*-value ≤ 0.05) between ASDs and controls at that given workload. ** marks a significant difference (*p*-value ≤ 0.05) in the increase from rest to peak exercise between the ASD and control group

At rest, parameters of LV systolic and diastolic function were comparable between groups apart from LV-s′ that was significantly lower for the ASD group. During exercise, LVEF, LV-GLS, LV-s′ and E/é gradually increased among controls (Fig. 3 and Table 3). In ASD patients, LVEF and LV-GLS remained unchanged during exercise, and peak LVEF exercise values were significantly lower compared with controls (*p* = 0.01). ASD patients demonstrated a lower increase in LVEF from baseline to peak exercise compared with controls (Fig. 3). LV-s′ increased during exercise in both groups but with a lower level at peak exercise in ASD patients compared with controls (Table 3).

### Right ventricular function

**Fig. 4:**
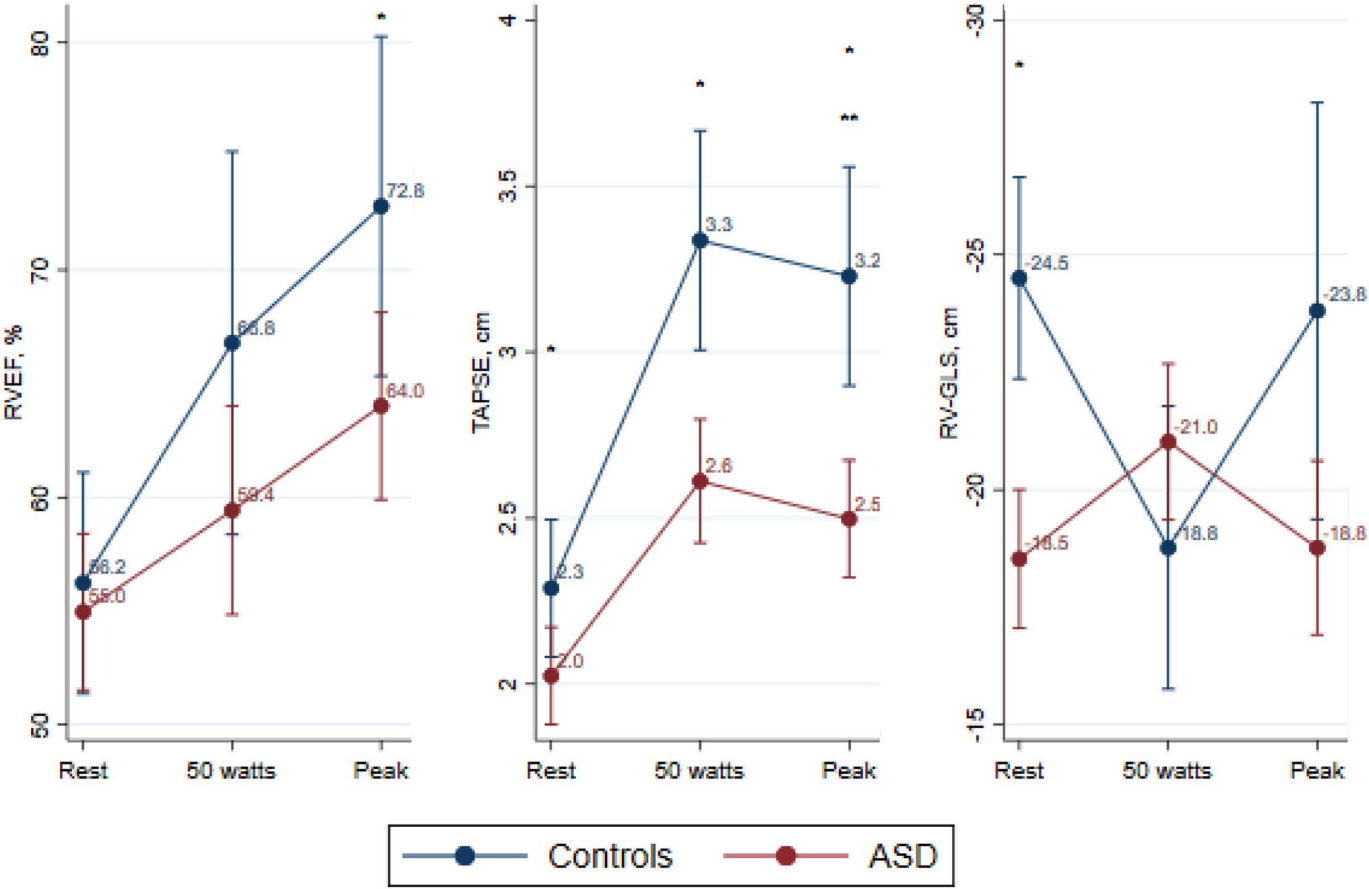
Right ventricular parameters. Mean and 95% confidence intervals at rest, exercise at 50 watts and peak exercise. Comparative analyses are performed using two-way mixed effects analysis of variance. *RVEF* Right ventricular ejection fraction, *TAPSE* tricuspid annular plane systolic excursion, *RV-GLS* Right ventricular global longitudinal strain. * marks a significant difference (*p*-value ≤ 0.05) between ASDs and controls at that given workload, ** marks a significant difference (p-value ≤ 0.05) in the increase from rest to peak exercise between the ASD and control group

RV systolic function evaluated by RVEF, RV FAC, TAPSE, and RV-s′ increased during exercise for both groups (Fig. 4 and Table 3). However, peak RVEF, peak RV FAC, and peak TAPSE were lower in ASD patients compared with controls, and the absolute increase in TAPSE from rest to peak exercise was significantly lower in the ASD group (Fig. 4). RV-GLS was lower in ASDs at rest but within normal range and comparable with controls during and at peak exercise.

Subgroup analyses showed that resting TAPSE was lower for the surgically corrected ASD patients (1.9 cm, 95% CI 1.7–2.0 cm) compared with the percutaneously corrected ASD patients (2.2 cm, 95% CI 2.0–2.4 cm, *p* = 0.02) and controls (2.3 cm, 95% CI 2.1–2.5 cm, *p* = 0.003). Peak TAPSE was similarly reduced in surgically corrected ASD patients (2.2 cm, 95% CI 2.0–2.5 cm) compared with percutaneously corrected patients (2.8 cm, 95% CI 2.5–3.0 cm, *p* = 0.003) and controls (3.2 cm, 95% CI 2.9–3.5 cm, *p* < 0.001). RV-s′ at rest was significantly lower in surgically corrected ASD patients (7.5 cm/s, 95% CI 6.9–8.2 cm/s) compared with percutaneously corrected ASDs (9.8 cm/s, 95% CI 9.1–10.5 cm/s, *p* < 0.001) and controls (10.2 cm/s, 95% CI 9.2–11.2 cm/s, *p* < 0.001). Peak RV-s′ was significantly lower in surgically corrected ASD patients (12.9 cm/s, 95% CI 11.7–14.2 cm/s) compared with controls (15.2 cm/s, 95% CI 13.6–16.8 cm/s, *p* = 0.04).

### Reproducibility

In this study, reproducibility was assessed for resting and peak LVEF and TAPSE. Reproducibility for peak LV GLS and resting RVEF have previously been assessed in our laboratory [19, 20].

Resting LVEF intraobserver variability yielded an absolute mean difference of 2.7 ± 4.1% and an intraclass correlation coefficient (ICC) of 0.90 (95% CI: 0.69–0.97), while interobserver variability yielded an absolute mean difference of 4.1 ± 4.6% and ICC of 0.86 (95% CI: 0.57–0.95). Peak LVEF intraobserver variability resulted in an absolute mean difference of 0.07 ± 4.1% and ICC of 0.91 (95% CI: 0.73–0.97) and interobserver variability resulted in an absolute mean difference of 1.5 ± 5.5% and ICC of 0.80 (95% CI: 0.39–0.93). Our laboratory has previously documented that peak exercise LV GLS had an absolute mean difference of 0.15% and ICC of 0.96 for intraobserver variability, and absolute mean difference of 0.18% and ICC of 0.88 for interobserver variability [19].

Resting RVEF showed an absolute mean difference of 0 ± 6% and ICC of 0.94 for intraobserver variability, and absolute mean difference of 3 ± 12% and ICC of 0.82 for interobserver variability [20]. For resting TAPSE, the intraobserver variability yielded an absolute mean difference of 0.03 ± 0.1 cm and ICC of 0.98 (95% CI: 0.93–0.99) and interobserver variability yielded an absolute mean difference of 0.05 ± 0.1 cm, and ICC of 0.97 (95% CI: 0.92–0.99). The intraobserver variability for peak TAPSE resulted in an absolute mean difference of 0.3 ± 0.3 cm and ICC of 0.83 (95% CI: 0.51–0.94) and interobserver variability resulted in an absolute mean difference of 0.3 ± 0.3 cm and ICC of 0.83 (95% CI: 0.50–0.94).

## Discussion

In the present study assessing patients with an isolated secundum ASD almost two decades after correction, we find: the biventricular systolic function at rest is comparable with controls; the LV systolic exercise response in terms of the expected increase of LVEF and LV-GLS is impaired and LVEF is significantly lower compared with controls at peak exercise; and finally, RV systolic function expressed by widely accepted parameters such as RVEF and TAPSE increased during exercise in both groups, but at peak exercise, these parameters were significantly lower in the ASD group, while RV-GLS was comparable during exercise.

Our results show that the corrected ASD patients are comparable with healthy controls with respect to maximal oxygen consumption at peak exercise. Therefore, one would expect that ASD patients have a correspondingly normal myocardial function at rest, during, and at peak exercise. In accordance with previous reports, the LV and RV systolic function were normal and comparable with controls at rest [4, 8]. However, at peak exercise, we demonstrate a reduced RV and, in particular, LV systolic capacity in ASD patients. As shown in other cardiac diseases, exercise stress echocardiography might reveal signs of subclinical myocardial dysfunction that go undetected during resting examinations [23–25]. An older study by Bonow et al.[13] showed an abnormal left ventricular systolic exercise response in uncorrected ASD patients. Our findings indicate that impaired systolic myocardial reserve capacity can be present in corrected ASD patients as well. The presence of subclinical myocardial dysfunction is in addition supported by a recently published invasive hemodynamic study from our group demonstrating RV and LV filling pressures were elevated at rest but particularly during physiological exercise [15]. Indications of abnormal ventricular filling conditions could be related to abnormal atrial compliance, atrial systolic function, but also abnormal LV myocardial properties. The present data support that a degree of abnormal latent LV and RV systolic dysfunction might be present.

Systolic function is traditionally evaluated by LVEF and RVEF, both derivated from volume changes expressing in particular the myocardial radial systolic function. We find both LVEF and RVEF at peak exercise to be reduced in ASD patients compared with controls. LV-GLS, a measure of LV longitudinal systolic function, is able to reveal subtle systolic myocardial dysfunction, especially in conditions with preserved LVEF [26, 27]. LV-GLS is considered normal when within a range of −15.9% to −22.1% at rest [28–30]. An increase of at least 5% from rest to peak exercise can be expected with a peak LV-GLS value of −25 ± 3% in normal subjects [19]. In the present study, the ASD group did not improve the LV longitudinal systolic function during exercise as expected. Similarly, Bussadori et al.[5] found that resting LV-GLS did not change when comparing pre- and post-corrective values. Furthermore, the systolic tissue Doppler parameter s´ measuring the lateral mitral annular velocity during systole were noted to have lower values in the ASD patients as compared with controls. Based on the consistent reduction of peak stress values of three well-accepted LV systolic function measures in the ASD group, the LV systolic response to physical stress is impaired implying signs of subtle myocardial dysfunction.

Strain measurements are also applicable to the RV. Normal average RV-GLS values have been reported to be around −26 ± 3%, but these values might not reflect the overall longitudinal systolic function of the RV [9]. The RV is a morphologically complex structure, and the evaluation of RV systolic deformation with strain analysis based solely on an apical modified 4-chamber view might be too simplified [31]. Despite these limitations, RV-GLS at rest is lower in ASD patients, yet not much different than what has been reported postoperatively by Bussadori et al.[5] and Jategaonkar et al.[8] We noted that RV-GLS did not change during exercise in either group. RV-GLS scores may reflect both RV and LV contractility. The myofibers in the RV wall continue into the LV at the apex, and both ventricles are connected through a common septum resulting in ventricular interdependence [31]. Therefore, it can be suspected that abnormalities in LV myocardial function impact RV myocardial function and are reflected in the RV-GLS values or vice versa. The “reverse Bernheim effect”, LV dysfunction as a result of RV volume or pressure overload, may be present prior to ASD correction. Once corrected, the RV overload is immediately removed, and the interventricular septum adjusts accordingly allowing for a greater filling of the LV. This acute LV volume loading may induce LV contractile dysfunction eventually causing heart failure [32]. Particularly in elderly patients with a long-standing left-to-right shunt and increased left atrial pressure as their LV may not readily adapt to an acute volume loading due to being underfilled for a long time period. For this reason, an ASD test occlusion could be performed to detect those at risk of post-corrective heart failure [33].

TAPSE and RV-s′ are also widely used measures of RV systolic function obtained from the lateral tricuspid annulus. TAPSE represents the tricuspid annular apical upward displacement and RV-s′ represents the lateral annular velocity and both parameters are considered to reflect the RV longitudinal systolic function. In asymptomatic uncorrected ASD patients, RV systolic parameters increase during exercise with peak RVEF and TAPSE exceeding peak values of control subjects [34]. This indicates that persistent physiological RV volume overload allows for a hyperdynamic RV systolic response to exercise. In our cohort, the corrected ASD patients’ RV systolic response to exercise is diminished; even though TAPSE increases during exercise, it is unfailingly lower compared with controls, a finding also supported by Lange et al.[7] TAPSE is consistently reported to be lower following open-heart surgery, and potentially also following percutaneous ASD closure [4, 7, 35–37]. Our results correlate with previous findings, as resting and peak TAPSE in surgically corrected ASD patients are lower than in percutaneously corrected ASD patients and controls. TAPSE should therefore be interpreted carefully, as it may not fully reflect RV systolic myocardial function, especially when other RV systolic parameters (e.g. RV-s′) indicate post-corrective improvements. RV-s′ in our ASD patients is reduced at rest yet comparable with the control cohort during exercise reflecting that the velocity of the tricuspid annulus is intact. Whether it actually reflects preserved RV systolic longitudinal function is opposed by the reduced values of RVEF, RV-GLS and RV FAC.

When comparing the echocardiographically evalauted myocardial function parameters between the percutaneously corrected and surgically corrected ASD patients, only TAPSE and RV-s′ differed between these two subgroups. This is believed to be related to the differences of their invasive treatment procedures as discussed above.

In this study, we demonstrate signs of an impaired RV and LV response to exercise in corrected ASD patients. The corrected ASD patients have preserved exercise capacity in terms of peak oxygen consumption suggesting that the present findings should be classified as subclinical abnormalities of myocardial function. In addition, our group has documented an abnormal hemodynamic response to exercise assessed by invasive right heart catheterization in this patient cohort as well [15]. Their atrial pressures were increased at both rest and peak exercise compared to the controls[15], however, they did not develop signs of pulmonary arterial hypertension even if this might occur post correction[38] nor did our patients have increased pulmonary vascular resistance. The data presented in this paper support abnormal myocardial properties in corrected ASD patients. Properties that may be linked to the long-term increased risk of morbidity including higher risk of early development of atrial fibrillation seen in ASD patients [10, 11]. The exact clinical implications of our findings cannot currently be determined, as this would require follow-up data showing progression of the physiological abnormalities or direct causality to the long-term risk outcomes. Regular examinations of this patient cohort may potentially allow us to identify at-risk patients and introduce medical treatment at an early stage rather than treating patients once they develop symptomatic risk outcomes such as arrhythmia or heart failure at which point their quality of life may already have decreased.

## Limitations

The study cohort is relatively small but was extensively examined at rest and during increasing levels of strain to describe the response to physiological stress. Not all invited ASD patients accepted the study invitation why the results may represent a certain unintentional selection bias.

Matching for sex was not possible due to available controls, which could have affected the reported results. Larsen et al.[19] documented that sex differences were not present for LV-GLS and LV diastolic parameters during exercise, whereas sex difference was present for LV-s′ with increasing exercise.When including subjects we were not able to control for pre-closure characteristics such as ASD size, shunt size, ventricular function and pressure. This may have influenced the results as the study cohort is relatively small. Additionally, we were unable to determine if myocardial capacity evaluated during exercise was correlated to ventricular function prior to correction.

RV-GLS measurements are subject to a certain degree of variation. Precise GLS measurements require high quality images visualizing at least 4 out of 6 wall segments, which may be a further challenge during physical exercise with increasing heart rates and might result in RV-GLS underestimation. Our RV-GLS measurements are based upon both septal and lateral wall segments (6-segment method). This is a more reliable method to assess RV-GLS rather than from the free wall only (3-segment method). As more wall segments are evaluated, poor imaging is less likely to affect the entire region of interest. However, RV-FWGLS is known to yield 5% higher strain values than RV-GLS estimation [9].

The RV values are extracted from 2D images, which are unable to fully embrace and visualize the complex RV morphology as 3D images. However, 3D imaging during cardiopulmonary exercise is not possible as stitching artifacts would occur in the images and render them unanalyzable.

## Conclusion

Corrected ASD patients demonstrate a reduced RV and LV systolic response to exercise decades after correction. Even though exercise capacity is preserved, an impaired increase in peak biventricular systolic parameters, such as ejection fraction and GLS, were noted in ASD patients compared with controls. This study indicates that ASD patients have signs of subclinical systolic myocardial dysfunction, which could potentially be linked to previously documented long-term morbidities.

### Supplementary Information

Below is the link to the electronic supplementary material.

Supplementary file1 (WMV 1801 kb)

Supplementary file2 (WMV 2379 kb)

Supplementary file3 (WMV 1731 kb)

## Data Availability

Data supporting this study's findings will be available from the corresponding author upon reasonable request and according to applicable legal and ethical requirements.

## Abbreviations

ASD: Atrial septal defect
BSA: Body surface area
CPX: Cardiopulmonary exercise test
ICC: Intraclass correlation coefficient
LA: Left atrium
LV: Left ventricle
LV-a′: Lateral mitral annular a’ wave velocity
LV-e′: Lateral mitral annular e’ wave velocity
LVEF: Left ventricular ejection fraction
LV-GLS: Left ventricular global longitudinal strain
LV-s′: Peak systolic lateral mitral annular velocity
RA: Right atrium
RER: Respiratory exchange ratio
RV: Right ventricle
RVEF: Right ventricular ejection fraction
RV FAC: Right ventricular fractional area chance
RV-FWGLS: Right ventricular free wall global longitudinal strain
RV-GLS: Right ventricular global longitudinal strain
RV-s′: Peak systolic tricuspid annular velocity
TAPSE: Tricuspid annular plane systolic excursion
VO_2_: Oxygen uptake
